# Decreased Thalamic Activity Is a Correlate for Disconnectedness during Anesthesia with Propofol, Dexmedetomidine and Sevoflurane But Not S-Ketamine

**DOI:** 10.1523/JNEUROSCI.2339-22.2023

**Published:** 2023-06-28

**Authors:** Oskari Kantonen, Lauri Laaksonen, Michael Alkire, Annalotta Scheinin, Jaakko Långsjö, Roosa E. Kallionpää, Kaike Kaisti, Linda Radek, Jarkko Johansson, Timo Laitio, Anu Maksimow, Joonas Scheinin, Mikko Nyman, Mika Scheinin, Olof Solin, Tero Vahlberg, Antti Revonsuo, Katja Valli, Harry Scheinin

**Affiliations:** ^1^Turku PET Centre, University of Turku and Turku University Hospital, Turku FI-20521, Finland; ^2^Department of Perioperative Services, Intensive Care and Pain Medicine, Turku University Hospital, University of Turku, Turku FI-20521, Finland; ^3^Department of Perioperative Services, Intensive Care and Pain Medicine, Satakunta Central Hospital, Pori FI-28500, Finland; ^4^Department of Anesthesiology and Perioperative Medicine, University of California, Irvine, Irvine, California 92868; ^5^Department of Intensive Care, Tampere University Hospital, Tampere FI-33521, Finland; ^6^Department of Psychology and Speech-Language Pathology, and Turku Brain and Mind Center, University of Turku, Turun yliopisto FI-20014, Finland; ^7^Department of Anesthesiology and Intensive Care, Oulu University Hospital, Oulu FI-90029, Finland; ^8^Department of Radiation Sciences, Umeå University, Umeå SE-901 87, Sweden; ^9^Department of Radiology, Turku University Hospital, Turku FI-20521, Finland; ^10^Institute of Biomedicine and Unit of Clinical Pharmacology, University of Turku and Turku University Hospital, Turun yliopisto FI-20014, Finland; ^11^Institute of Clinical Medicine, Biostatistics, University of Turku and Turku University Hospital, Turun yliopisto FI-20014, Finland; ^12^Department of Cognitive Neuroscience and Philosophy, School of Bioscience, University of Skövde, Skövde SE-541 28, Sweden

**Keywords:** Anesthesia, connected, consciousness, disconnected, neuroimaging, positron emission tomography

## Abstract

Establishing the neural mechanisms responsible for the altered global states of consciousness during anesthesia and dissociating these from other drug-related effects remains a challenge in consciousness research. We investigated differences in brain activity between connectedness and disconnectedness by administering various anesthetics at concentrations designed to render 50% of the subjects unresponsive. One hundred and sixty healthy male subjects were randomized to receive either propofol (1.7 μg/ml; *n* = 40), dexmedetomidine (1.5 ng/ml; *n* = 40), sevoflurane (0.9% end-tidal; *n* = 40), S-ketamine (0.75 μg/ml; *n* = 20), or saline placebo (*n* = 20) for 60 min using target-controlled infusions or vaporizer with end-tidal monitoring. Disconnectedness was defined as unresponsiveness to verbal commands probed at 2.5-min intervals and unawareness of external events in a postanesthesia interview. High-resolution positron emission tomography (PET) was used to quantify regional cerebral metabolic rates of glucose (CMR_glu_) utilization. Contrasting scans where the subjects were classified as connected and responsive versus disconnected and unresponsive revealed that for all anesthetics, except S-ketamine, the level of thalamic activity differed between these states. A conjunction analysis across the propofol, dexmedetomidine and sevoflurane groups confirmed the thalamus as the primary structure where reduced metabolic activity was related to disconnectedness. Widespread cortical metabolic suppression was observed when these subjects, classified as either connected or disconnected, were compared with the placebo group, suggesting that these findings may represent necessary but alone insufficient mechanisms for the change in the state of consciousness.

**SIGNIFICANCE STATEMENT** Experimental anesthesia is commonly used in the search for measures of brain function which could distinguish between global states of consciousness. However, most previous studies have not been designed to separate effects related to consciousness from other effects related to drug exposure. We employed a novel study design to disentangle these effects by exposing subjects to predefined EC_50_ doses of four commonly used anesthetics or saline placebo. We demonstrate that state-related effects are remarkably limited compared with the widespread cortical effects related to drug exposure. In particular, decreased thalamic activity was associated with disconnectedness with all used anesthetics except for S-ketamine.

## Introduction

Anesthetic drugs cause reversible changes in the state of consciousness and in responsiveness to external stimuli, but the underlying neural mechanisms remain incompletely understood (Scheinin et al., 2018a). Changes in multiple domains of brain function between responsive baseline and anesthetic-induced unresponsive conditions have been studied extensively and are relatively well characterized for most, commonly used anesthetics (Bonhomme et al., 2019). Yet, a central limitation in most studies exploring the neural mechanisms by which anesthetics induce changes in the global state of consciousness is the difficulty to point out which of the observed changes in neural function contribute to the altered state of consciousness (i.e., state-related effects), and which may reflect other effects caused by the administered anesthetics (i.e., nonspecified drug-effects; Scheinin et al., 2018b). However, a growing body of evidence from our group (Långsjö et al., 2012; Scheinin et al., 2018b, 2021; Kallionpää et al., 2020) and others (Xie et al., 2011; Pal et al., 2020; Casey et al., 2022) indicates that it is possible to dissociate these effects by careful study design choices, and suggests that more limited changes in neural function associate with the state-effect, than what can be observed if the nonspecified drug-effects are not controlled for.

In our recent study (Scheinin et al., 2021), we used forced awakenings during steady-state propofol and dexmedetomidine anesthesia to dissociate state-related effects from nonspecified drug-effects in a within-subject design. Positron emission tomography (PET) imaging with [^15^O]H_2_O tracer revealed that the transitions between disconnected and connected states of consciousness were consistently associated with concomitant reciprocal changes in brain activity primarily in the thalamus, anterior and posterior cingulate cortices and angular gyri. Notably, the pattern separating these states was markedly different from the widespread pattern of suppressed cortical activity commonly associated with anesthetic-induced unresponsiveness (Kaisti et al., 2002; Laitio et al., 2009; Akeju et al., 2014).

While the serial awakening paradigms have previously been implemented to reveal neural mechanisms related to specific states of consciousness, the studies have been limited to using propofol and dexmedetomidine as anesthetics. The threshold-level dosing required in such experiments has proven problematic with other commonly used anesthetics, such as ketamine and sevoflurane, because of excessive cognitive impairment (Casey et al., 2022) or movement in the scanner (Palanca et al., 2015). Hence, to study the state-related effects of a wide range of anesthetics with different primary mechanisms of action, we randomized healthy male volunteers to receive EC_50_ for unresponsiveness to verbal command of either propofol, dexmedetomidine, sevoflurane or S-ketamine using a parallel-group design and applied PET imaging with ^18^F-fluorodeoxyglucose (FDG) tracer to measure brain activity. Trapping of the tracer inside active brain cells creates a long-lasting imprint of the distribution of metabolic activity in the brain during the drug intervention, enabling brain scans to be acquired later when the subjects are awake and co-operative, rather than during the anesthesia (Alkire et al., 1999). A placebo group was included to allow comparisons between the drug-free wakeful state and anesthesia.

The study was designed to answer three main questions: (1) what are the neural mechanisms of disconnectedness, assessed by contrasting scans from subjects in different states of consciousness but with similar drug exposure; (2) are these state-related mechanisms shared across different anesthetics when the drug-effect is accounted for; and (3) what are the nonspecified drug-effects and combined effects of the drug and the state? Connectedness is here defined as the ability to consciously perceive external stimuli and to either perform meaningful voluntary behavioral responses or later report having experienced external events. Disconnectedness may indicate total absence of internal conscious experiences, but is also consistent with the presence of internally generated, externally disconnected conscious experiences (e.g., dreams).

## Materials and Methods

### Subjects

One hundred and sixty 18- to 30-year-old healthy, ASA 1 (according to the American Society of Anesthesiologists physical status classification system), right-handed volunteers participated in the study. The trial (NCT0262440) was approved by the Ethics Committee of the Hospital District of Southwest Finland and the Finnish Medicines Agency Fimea. All subjects provided written informed consent in accordance with the Declaration of Helsinki. Radiation exposure concerns related to the PET imaging limited the subjects to only male volunteers. All subjects were interviewed and examined by a licensed physician (L.L., A.S., or O.K.). A standard 12-lead electrocardiogram (ECG) and blood and urine samples were also taken. Exclusion criteria included any somatic illness, chronic medication or drug allergy, history of any psychiatric disorder or substance abuse, cardiac arrhythmias, hearing impairment, serious nausea in connection with previous anesthesia, blood donation within 90 d before the study, prior participation in a PET/SPECT study, any contraindication to magnetic resonance imaging (MRI), and pathologic findings in laboratory tests or positive urine drug screening.

### Experimental design

**Figure 1.**
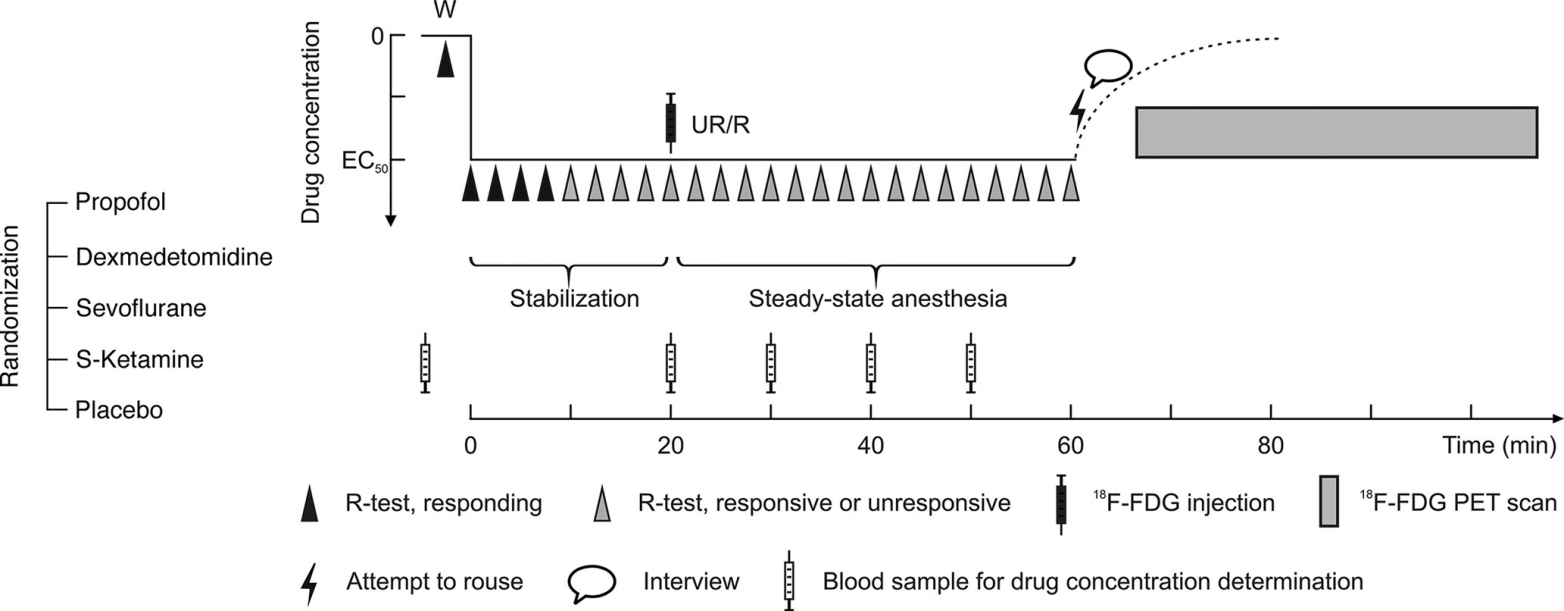
Design of the experiment. Each subject within each treatment group received a targeted EC_50_ concentration of either propofol, dexmedetomidine, sevoflurane, or S-ketamine. After a 20-min stabilization phase, FDG tracer was injected followed by a 40-min steady-state anesthesia phase, allowing brain FDG uptake to be completed. Then, administration of the study treatment was terminated, and an attempt was made to rouse the unresponsive subjects, followed by a brief interview to probe for mental content during the anesthesia. Next, the subjects were moved to the PET scanner for brain imaging. Responsiveness was tested every 2.5 min during the steady-state anesthesia. Behavioral states of interest: W = wakeful baseline, UR = unresponsive, R = responsive. See the Materials and Methods section for details.

We used the PET neuroimaging data, responsiveness test data and structured interview data from our recently published studies (Laaksonen et al., 2018; Radek et al., 2021) to investigate differences in cerebral metabolic rates of glucose (CMR_glu_), as a surrogate measure for neuronal activity (Varrone et al., 2009), between connectedness and disconnectedness, and between exposure to anesthetic agents and placebo. The design of the study is schematically illustrated in Figure 1.

Based on our previous studies, we estimated a target concentration which would render 50% of the subjects unresponsive to verbal command, i.e., EC_50_, for each study drug. The selected EC_50_ target values were: 1.7 µg ml^−1^ for propofol, 1.5 ng ml^−1^ for dexmedetomidine, 0.9% end-tidal for sevoflurane and 0.75 µg ml^−1^ for S-ketamine (Långsjö et al., 2005, 2012; Kaskinoro et al., 2011; Scheinin et al., 2021). The subjects received individually programmed, targeted EC_50_ infusions (or end-tidal %) of either propofol (*n* = 40), dexmedetomidine (*n* = 40), sevoflurane (*n* = 40), S-ketamine (*n* = 20), or saline placebo (*n* = 20). The participants were randomly allocated to the different treatments, but the treatments were not masked. Permuted blocks were applied to achieve balanced groups across treatments. The anesthetic protocol was initiated with a 20-min stabilization phase, after which the FDG PET radiotracer was injected, and the achieved pseudo-steady-state phase was maintained for 40 min. Responsiveness to verbal command was tested every 2.5 min during anesthesia and arterial blood samples were drawn for drug concentration measurements and tracer kinetic modeling. Brain retention of the FDG tracer was considered to be stabilized after the 40 min pseudo-steady-state anesthesia phase (Alkire et al., 1997). During this period, the FDG tracer was taken up to metabolically active brain cells and converted to [^18^F]FDG-6-phosphate via hexokinase-mediated phosphorylation (Duelli and Kuschinsky, 2001; Varrone et al., 2009). Because [^18^F]FDG-6-phosphate cannot be metabolized further and has low membrane permeability, it becomes trapped inside the cells with a very slow clearance (Gallagher et al., 1978; Phelps et al., 1979). Because of these tracer properties, the subsequent PET scan effectively reflects glucose metabolism in the brain during the preceding tracer uptake period (Alkire et al., 1999). After terminating the administration of the treatment, subjects were awakened (if unresponsive), and a detailed interview of subjective experiences was conducted for all subjects. Finally, a PET scan measuring absolute CMR_glu_ was acquired. One scan and one interview were collected for each subject.

### Anesthesia protocol

The subjects abstained from the use of alcohol and any medications for at least 48 h and from the use of caffeine-containing products for 10–12 h and fasted overnight before the experiments. Two forearm veins were cannulated for administration of study treatments and the PET radiotracer. The left radial artery was cannulated to obtain blood samples for drug concentration measurements and kinetic modeling of the PET radiotracer. The intravenous anesthetic agents, propofol (Propofol Lipuro 10 mg/ml, B. Braun), dexmedetomidine (Dexdor 100 μg/ml, Orion Pharma) and S-ketamine (Ketanest-S 25 mg/ml, Pfizer) were administered using target controlled infusion (TCI) with previously described pharmacokinetic parameters (Domino et al., 1984; Marsh et al., 1991; Talke et al., 2003). A Harvard 22 syringe pump (Harvard Apparatus) and a portable computer running Stanpump software (by Steven L. Schafer, MD; www.opentci.org/code/stanpump) were used for drug administration. Plasma target levels were used for the intravenous anesthetics. Sevoflurane (Sevorane 100%, AbbVie) was administered through a tight-fitting face mask using a standard anesthesia workstation (Kaskinoro et al., 2011) with fresh gas flow set at 6 l min^−1^.

### Responsiveness test and classification of the state of consciousness

During the experiment, the subjects were lying on bed with custom-made response handles secured to their wrists. Responsiveness was tested with a standardized prerecorded auditory request “press the handles twice” (R-test). Responding was practiced before the anesthesia. The R-test was presented with Presentation 17.0 stimulus delivery and experimental control software system (Neurobehavioral Systems Inc), and stimuli were delivered via headphones. The request was presented once at 2.5-min intervals throughout the experiment. A response to the R-test categorized the subject as responsive for the subsequent 2.5-min period. No response to R-test resulted in the subject being classified as unresponsive for the subsequent 2.5-min period.

Targeting an EC_50_ dose might imply that 50% of subjects will be unresponsive during the entire PET tracer uptake time and that 50% would remain responsive. Alternatively, attempting to hold subjects on the borderline of being conscious of external events, one might expect fluctuations between states of responsiveness could occur one or more times during the tracer uptake period (McKinstry-Wu et al., 2019; Wasilczuk et al., 2020). Hence, the categorization of each subject as responsive or unresponsive may depend in part on how much time each person spent in the predominant state of responsiveness. Furthermore, since the overall rate of tracer uptake in active brain cells varies as a function of tracer concentration in plasma, the time period right after the tracer injection (when the tracer concentration in the plasma is high) will influence the subsequent PET image more than the time periods toward the end of the experiment (when the tracer concentration in plasma is low). To address this, we modeled the relative FDG brain uptake for each R-test period using blood samples from the placebo group drawn during the experiment for tracer kinetic modeling. Based on this model, a weight was assigned to each R-test period reflecting their proportional significance in forming the final CMR_glu_ image used for statistical testing. For each subject, the values of individual R-test weights were then summed for both responsive and unresponsive trials to form estimations of total FDG uptake, and subsequently PET imaging signal in each respective state of responsiveness. Greater than 0.8× total FDG brain uptake while unresponsive (UR) during the 40-min steady-state drug exposure was chosen as a threshold to assign a subject to this behavioral category. Greater than 0.4 × total FDG uptake in the responsive (R) state was chosen as a threshold to assign a subject to this category. In case a subject would not qualify for either category (i.e., the state was too labile for the subject to be categorized as responsive or unresponsive) they would be assigned to a third category, unclassified (UC). Note that the classification of the subjects into responsiveness classes differs here from Radek et al. (2021), where the subjects were classified as responsive or unresponsive based on the last two R-test results for optimization of interview analyses. Here, the classification procedure was optimized for PET analysis so that it would best reflect the amount of tracer uptake (and thus PET imaging signal) in each responsiveness category.

The categorization of subjects as connected or disconnected was based on (1) responsiveness classification and (2) structured interviews conducted at the end of the treatment period (Radek et al., 2021). Subjects classified as responsive were considered connected and conscious. Subjects classified as unresponsive with no explicit report of external perceptual content in the structured interview were considered disconnected.

### Magnetic resonance imaging (MRI)

For each subject, an anatomic MRI scan of the brain (T1, T2, FLAIR) was performed before the PET imaging session for subsequent image preprocessing and exclusion of any brain anomalies. MRI scans were obtained with a Philips Ingenuity PET-MR 3T scanner (Philips Medical Systems) with the following scan parameters: TR = 25 ms, TE = 4.6 ms, flip angle 30°, scan time 376 s. A trained neuroradiologist (MN) evaluated the anatomic images for any pathologic findings.

### Positron emission tomography (PET) imaging

PET imaging was performed using an ECAT HRRT scanner (Siemens CTI; Eriksson et al., 2002). The HRRT is a dual-layer, LSO-LYSO crystal-detector scanner characterized by a nearly isotropic 2.5-mm intrinsic spatial resolution. In the reconstructed images spatial resolution is 2.5–3 mm in the radial and tangential directions and 2.5–3.5 mm in the axial direction in the 10 cm field-of-view covering most of the brain (de Jong et al., 2007). Subjects were positioned supine in the scanner, wearing an individual thermoplastic mask to minimize head movements. Head motion was monitored with a high-precision, stereotaxic tracking device (Polaris Vicra, Northern Digital).

^18^F was produced by irradiating enriched [^18^O]H_2_O with protons (cyclotron CC 18/9, D.V. Efremov Institute, St. Petersburg, Russia) and an automated device (Fastlab; GE Healthcare) was used in [^18^F]FDG synthesis according to Good Manufacturing Practice regulations. Radiochemical purity of the product exceeded 95%. A 300 MBq dose of [^18^F]FDG was administered over 15 s by an automated infusion system (Rad Injector, Tema Sinergie). After the tracer uptake period, administration of the study treatment was terminated, and a 30-min PET scan was performed followed by a CT transmission scan. PET data acquisition was initiated ∼45–50 min after tracer injection in list mode format. Images were formed using an iterative OP-OSEM algorithm with resolution modeling (12 iterations, 16 subsets, including corrections for attenuation, scatter, and random events). PET data were histogrammed into a single 30-min frame reflecting the assumption of stable [^18^F]FDG retention over this late time window. In case the measured head movement exceeded 2.5 mm, scans were divided into subframes and subsequently co-registered into a single 30 min frame by using a multiple acquisition frame image reconstruction procedure and an attenuation correction realignment algorithm (Johansson et al., 2016). This procedure was used in four dexmedetomidine scans, two S-ketamine scans, and two sevoflurane scans.

### Drug concentration measurements

Arterial blood samples for drug concentration analysis in plasma were drawn at baseline and at 20, 30, 40, and 50 min after the start of the administration of the intravenous anesthetics. High-performance liquid chromatography with tandem mass spectrometry (HPLC-MS/MS) was used for dexmedetomidine and S-ketamine. Propofol concentrations were measured with HPLC and fluorescence detection. Interassay coefficients of variation in the relevant concentration ranges were 1.2–2.9%, 3.7–8.0%, and 0.7–2.2%, respectively (Laaksonen et al., 2018). Online end-tidal anesthetic gas concentration monitoring was used for sevoflurane.

### PET data preprocessing

Voxel-wise maps of CMR_glu_ were generated on the basis of average [^18^F]FDG retention at 50–80 min after tracer injection and plasma radioactivity data. Individual MRIs were co-registered with the subjects' parametric brain images using SPM8. Voxel-wise maps of CMR_glu_ were calculated according to the following equation:

$${\text{CMR}}_{\text{glu}}=\frac{\text{FUR }\times {\text{ C}}_{\text{glu}}}{\text{LC}},$$

where FUR is the fractional uptake rate of [^18^F]FDG relative to the integral (0–90 min) of the [^18^F]FDG concentration in plasma, C_glu_ is the plasma glucose concentration, and LC is the lumped constant (0.65; Wu et al., 2003). Assuming an average brain tissue density of 1.04 g ml^−1^, CMR_glu_ values were converted into units of µmol 100 g^−1^ min^−1^.

Nonlinear mapping from the MRI to the MNI standard space was estimated using unified segmentation in SPM8, and the deformations were subsequently applied to the MRI and co-registered PET images. All normalized PET images were smoothed using an isotropic Gaussian kernel of 12-mm FWHM.

### Statistical analysis

The Kolmogorov–Smirnov test was applied to test the normality of the outcome variables. Pearson's χ^2^ test (χ^2^) was used to compare responsiveness between the treatments followed by *post hoc* χ^2^ tests between treatment pairs. The Kruskal–Wallis *H* test (*H*) with the Dwass, Steel, Critchlow-Flinger method in pairwise comparisons was used to compare simulation-based FDG uptake weights for subjects assigned to the responsive or unresponsive categories between the treatments. The mean measured drug concentrations between the responsive and unresponsive subjects was compared with two-sample *t* test. Voxel-averaged regional CMR_glu_ values were extracted within a 2-mm sphere region of interest (ROI) centered at the peak effect location from the SPM conjunction analysis (see below) using the MarsBar toolbox (Brett et al., 2002). Two-way ANOVA was used to analyze differences in absolute ROI CMR_glu_ values between the connected and disconnected subjects followed by contrasts within the drugs. Pearson correlation coefficients (*r*) and coefficients of determination (*R*^2^) were calculated to assess the associations of the modeling-based weights for UR and mean measured drug concentrations with the CMR_glu_ from the previously described ROI. When applicable, Bonferroni correction was used to correct for multiple comparisons. A two-tailed *p* < 0.05 was considered statistically significant. Results are given as means (SD) if not otherwise stated. Statistical analyses, except for the voxel-wise neuroimaging data analyses (see below), were performed with SAS System for Windows, version 9.4. (SAS Institute Inc.).

A flexible factorial model was constructed in SPM12 with treatment group and state (connected or disconnected) as factors. Two-way interactions between factors were modeled in the design matrix, and t-contrasts were generated to assess differences in regional brain activity between subjects classified as connected versus disconnected (i.e., state-related effects) within each treatment group. Conjunction analysis was used to investigate common and jointly significant state-related effects across selected treatments (Friston et al., 2005). Combined effects of drug and state were investigated by contrasting placebo with the disconnected state, and drug-effects only, by contrasting placebo with the connected state. Because of the *ad hoc* nature of the thresholds of simulated FDG uptake weight by which the subjects were assigned to behavioral categories R, UR, or UC, a supplementary continuous analysis using a multiple regression model with simulated uptake weights for the UR condition as covariates was conducted. Thus, as the UR weight reflects the proportion of total FDG uptake in the disconnected state, the regression analysis tests for significant associations between disconnectedness and regional CMR_glu_. Global normalization with proportional scaling was applied to account for interindividual variability in CMR_glu_ and global effects of anesthetics (Alkire et al., 2000). Clusters of voxels at *p* < 0.05 FWE corrected were considered as significant, starting with an initial voxel-level threshold of *p* < 0.001. Results are presented at a significance threshold of *p* < 0.001, uncorrected, in case the findings did not survive the correction for multiple comparisons, but were localized within regions previously associated with consciousness-related metabolic effects (Alkire et al., 2000; Långsjö et al., 2005). Because of increased statistical power in the conjunction analyses, a more stringent threshold of *p* < 0.05 FWE corrected at voxel level was used to allow localization of peak effects. Conjunction analyses were tested against the global null hypothesis. MRIcroGL (https://www.nitrc.org/projects/mricrogl/) and Surfice (https://www.nitrc.org/projects/surfice) were used for visualization. The Human Brainnetome Atlas (Fan et al., 2016) and the Atlas of the Human Brain (Mai et al., 2016) were used for localization of the findings in the MNI space.

## Results

### Behavioral state, subjective reports, and ensuing classification of the state of consciousness

**Figure 2.**
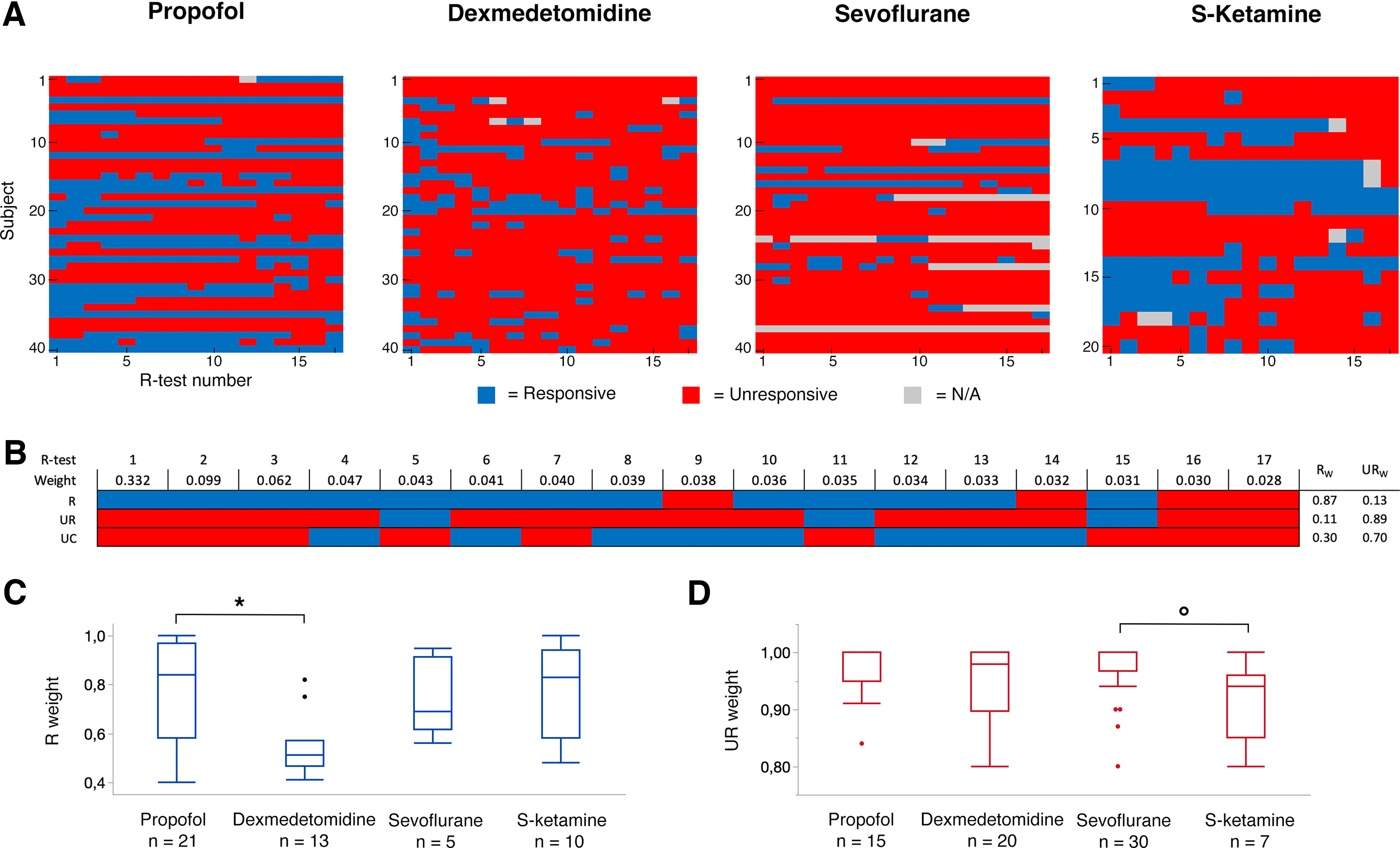
Responsiveness during the experiment. ***A***, Results of responsiveness tests performed every 2.5 min during the 40-min tracer uptake period and estimated EC_50_ steady state administration of each study drug. ***B***, Tracer kinetic modeling-based weights for each 2.5-min R-test period are displayed above R-test results from one representative subject for each responsiveness category. The weights were combined with R-test results to form an estimation of the proportion of total tracer uptake, and subsequently PET image signal, in either responsive (R_w_) or unresponsive (UR_w_) state as the sum of weights in each respective state (for details, see Extended Data Fig. 2-1). ***C***, ***D***, Boxplots of cumulative R-test weights for subjects classified as responsive (blue boxplots) or unresponsive (red boxplots) in each active treatment group. The median R weight of subjects classified as responsive was lower in the dexmedetomidine group than in the other treatment groups, but only reached statistical significance in pairwise comparison with the propofol group (*p = 0.0155). The median UR weight of subjects classified as unresponsive was significantly lower in the S-ketamine group compared with the sevoflurane group (°*p* = 0.0153). Boxes represent lower quartiles, medians and upper quartiles, and whiskers represent 1.5× interquartile ranges above and below the upper and lower quartiles. N/A, missing responsiveness data.

The subjects were prone to fluctuation in responsiveness because of the administered EC_50_ anesthetic dose, and they tended to become more unresponsive toward the end of the drug exposure phase (Fig. 2*A*). Weights based on tracer uptake kinetic modeling (Fig. 2*B*) were applied to assign subjects to responsiveness classes (R, UR, or UC; see Materials and Methods and Extended Data Fig. 2-1 for details). The final number of achieved states and cumulative model-based uptake weights for each active treatment group are presented in Table 1. The proportions of responsive, unresponsive, and unclassified subjects were significantly different in the sevoflurane group compared with the propofol and S-ketamine groups (*p* = 0.0024 and *p* = 0.016; Table 1). The median uptake weights for subjects classified as responsive were significantly different between the propofol and dexmedetomidine groups (*p* = 0.0115; Table 1; Fig. 2*C*). The median uptake weights for subjects classified as unresponsive were significantly different between the sevoflurane and S-ketamine groups (*p* = 0.0153; Table 1; Fig. 2*D*). Extended Data Figure 2-1 summarizes the R-test results in each study subject during the experiment, the calculated FDG brain uptake weight and the ensuing classification.

**Table 1.** Responsiveness state categorization and cumulative tracer uptake weights

Drug	Responsive		Unresponsive		Unclassified	
	*n* (%)	Uptake weight in responsive state	*n* (%)	Uptake weight in unresponsive state	*n* (%)	Uptake weight in responsive state
Propofol *n* = 40	21 (52.5%)	0.84 (0.58–0.94)	15 (37.5%)	1.00 (0.95–1.00)	4 (10%)	0.31 (0.27–0.33)
Dexmedetomidine *n* = 40	13 (32.5%)	0.51 (0.49–0.57)	20 (50%)	0.98 (0.90–1.00)	7 (17.5%)	0.37 (0.33–0.38)
Sevoflurane *n* = 40	5 (12.5%)	0.69 (0.67–0.88)	30 (75%)	1.00 (0.97–1.00)	5 (12.5%)	0.13 (0.07–0.19)
S-ketamine *n* = 20	10 (50%)	0.83 (0.61–0.94)	7 (35%)	0.94 (0.85–0.96)	3 (15%)	0.33 (0.25–0.34)

The final number (proportions) of subjects assigned to responsiveness state categories and median (IQR) uptake weights for respective categories in each treatment group. The overall proportions of responsive, unresponsive, and unclassified subjects were significantly different between treatment groups (χ^2^(6) = 18.4, *p* = 0.0052). In pairwise comparisons, the corresponding proportions were significantly different in the sevoflurane group compared with the propofol and S-ketamine groups (χ^2^(2) = 15.0, *p* = 0.0024) and (χ^2^(2) = 11.0, *p* = 0.016), respectively. There was a significant difference in median uptake weights between treatment groups in both responsive and unresponsive subjects (*H*(3) = 11.9, *p* = 0.0078) and (*H*(3) = 9.8, *p* = 0.0204), respectively. In pairwise comparisons, the difference was significant between propofol and dexmedetomidine groups in responsive subjects (*Z* = 3.1, *p* = 0.0115, corrected with the Dwass, Steel, Critchlow-Flinger method) and between sevoflurane and S-ketamine groups in unresponsive subjects (*Z* = 3.0, *p* = 0.0153, corrected with the Dwass, Steel, Critchlow-Flinger method).

10.1523/JNEUROSCI.2339-22.2023.f2-1 Extended Data Figure 2-1 Responsiveness weights based on tracer kinetic modeling and summary of responsiveness during the experiment. ***A***, Tracer kinetic modeling was used to estimate cumulative [^18^F]FDG brain uptake for each 2.5-min R-test period. Assuming stabilized brain retention of the [^18^FFDG tracer by the time of the last R-test, cumulative tracer uptake ratios were calculated for each R-test period by dividing the simulated uptake value at respective time frames with the total tracer uptake value. Weights were then obtained by calculating the increase in cumulative uptake ratio for every sequential R-test period, reflecting their proportional significance in forming the final CMR_glu_ image. ***B***, R-test results in each study subject measured at 2.5-min intervals. The weights were combined with R-test results to estimate total [^18^FFDG brain uptake in either a responsive or an unresponsive state. Final weights and the ensuing subject classification (UR, R, or UC) are indicated in the three columns to the right. UR = unresponsive, R = responsive, UC = unclassified. Download Figure 2-1, TIF file.

After drug administration, 11 (out of 15), 18 (out of 20), and 11 (out of 30) unresponsive subjects from the propofol, dexmedetomidine and sevoflurane groups, respectively, were successfully interviewed for mental content during the preceding unresponsive period. Disconnected experiences, i.e., experiences taking place during unresponsiveness but not directly associated with external perceptual content, such as dreams, were reported by 4/11 (36%), 15/18 (83%), and 5/11 (45%) of subjects in the propofol, dexmedetomidine and sevoflurane groups, respectively. None of the unresponsive subjects in the S-ketamine group could be interviewed immediately after terminating the drug infusion because of residual drug-effects. There were no reports indicating connectedness. Thus, all unresponsive subjects were classified as disconnected, whereas all responsive subjects were classified as connected and conscious.

### Drug exposure

There were no statistically significant differences between measured drug concentrations in plasma (or end-tidal air) between the connected and disconnected subjects within the treatment groups (*p* > 0.05; Table 2). Thus, any differences in brain metabolism between connected and disconnected subjects may be considered to be related to the difference in the compared states.

**Table 2.** Targeted and measured drug concentrations during the experiment

Drug	Targeted concentration	Responsive	Unresponsive	Two-sample *t* test
		Measured	Measured	
Propofol (μg/ml)	1.7 (0.0)	1.65 (0.35)	1.89 (0.33)	*t*_(34)_ = −2.06 *p* = 0.190
Dexmedetomidine (ng/ml)	1.5 (0.0)	2.09 (0.40)	2.16 (0.38)	*t*_(31)_ = −0.53 *p* = 1.00
Sevoflurane (% end-tidal)	0.90 (0.0)	0.90 (0.01)	0.91 (0.03)	*t*_(33)_ = −1.22 *p* = 0.919
S-ketamine (μg/ml)	0.75 (0.0)	0.93 (0.15)	1.05 (0.20)	*t*_(15)_ = −1.41 *p* = 0.720

Mean (SD) targeted and measured drug concentrations in plasma or end-tidal gas in the propofol, dexmedetomidine, sevoflurane and S-ketamine groups. No statistically significant differences were observed in the measured concentrations between the responsive and unresponsive subjects (two-tailed *p* > 0.05, Bonferroni corrected).

### State-related effects

**Figure 3.**
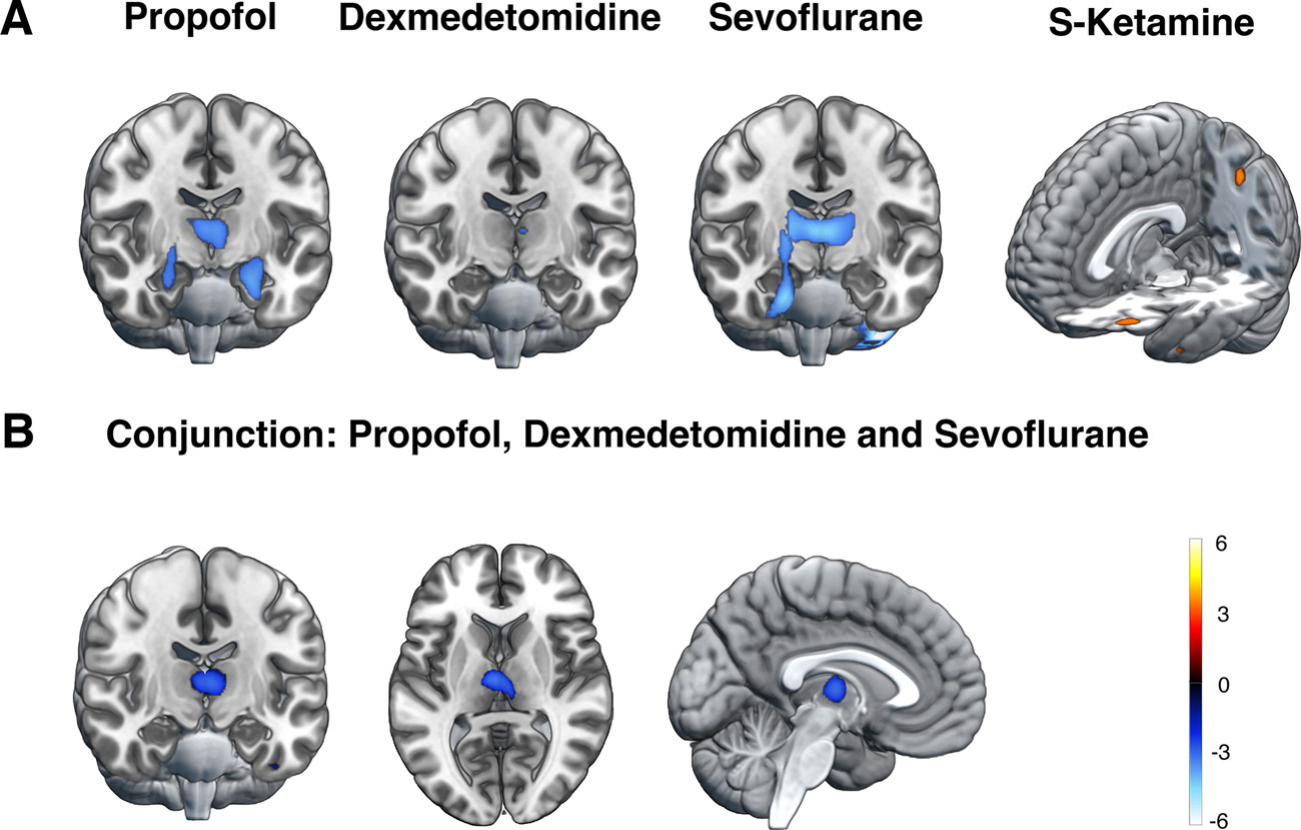
State-related effects. ***A***, Relative CMR_glu_ differences between connectedness and disconnectedness within each treatment group representing state-related effects for each drug. Cool colors represent reduced, and warm colors increased relative glucose metabolism in the disconnected state as contrasted to the connected state. In the propofol and sevoflurane groups, decreased thalamic and medial temporal cortex activity was associated with disconnectedness (*p* < 0.05, FWE corrected at cluster level). In the dexmedetomidine group, a state-related effect of decreased activity was found in the left midline thalamus (*p* < 0.001, uncorrected). In the S-ketamine group, disconnectedness was associated with increased activity at the intersection of the left intraparietal sulcus and dorsal angular gyrus (*p* < 0.001, uncorrected). ***B***, A conjunction analysis of the contrasts between connectedness and disconnectedness across propofol, dexmedetomidine and sevoflurane groups revealed a significant overlapping effect of decreased CMR_glu_ in the absence of connected consciousness bilaterally in the thalamus, with largest reductions in the left central medial thalamic nucleus (*p* < 0.05, FWE corrected at voxel level). The colorbar depicts *t* values. Similar results were obtained in a regression analysis using each subject's UR weight (a measure of disconnectedness) as a regressor and brain metabolism as a response variable (Extended Data Fig. 3-1).

In the propofol group, disconnectedness was associated with relatively lower glucose metabolism bilaterally in the thalamus, hippocampus, amygdala, nucleus accumbens, nucleus caudatus, and globus pallidus (*p* < 0.05, FWE corrected at cluster level; Fig. 3*A*). Within the sevoflurane group, similar state-related CMR_glu_ differences were found bilaterally in the thalamus and the cerebellum, and in the right hippocampus, amygdala, anterior parahippocampal gyrus, temporal fusiform cortex and globus pallidus. In the dexmedetomidine and S-ketamine groups, no statistically significant differences were found after correcting for multiple comparisons. However, with a significance threshold of *p* < 0.001, uncorrected, disconnectedness was associated with lower activity in the left midline thalamus in the dexmedetomidine group, and higher activity at the intersection of the left intraparietal sulcus and angular gyrus in the S-ketamine group.

10.1523/JNEUROSCI.2339-22.2023.f3-1 Extended Data Figure 3-1 Relationship between disconnectedness and CMR_glu_. ***A***, In the propofol group, disconnectedness was negatively correlated with CMR_glu_ bilaterally in the thalamus (*p* < 0.05, FWE corrected at cluster level) In the sevoflurane group, similar correlations were found bilaterally in the thalamus, right medial temporal cortex, and the cerebellum. In the dexmedetomidine and S-ketamine groups, state-metabolism correlations were found only at a significance threshold of *p* < 0.001, uncorrected. In the dexmedetomidine group, disconnectedness was negatively correlated with CMR_glu_ in the left medial thalamus and ventral posterior cingulate cortex, while in the S-ketamine group, there was a positive correlation between disconnectedness and CMR_glu_ at the intersection of the left intraparietal sulcus and dorsal angular gyrus and in the left rostroventral angular gyrus. ***B***, Conjunction analysis across propofol, dexmedetomidine and sevoflurane groups revealed overlapping negative correlation between disconnectedness and CMR_glu_ mainly bilaterally in the thalamus, and to lesser extent bilaterally in the inferior temporal cortices and dorsal insular cortex (*p* < 0.05, FWE corrected at voxel level). The colorbar depicts *t* values. Download Figure 3-1, TIF file.

Next, we used conjunction analysis to investigate consistent effects related to disconnectedness across treatments. Since the within-treatment group comparisons suggested different consciousness-related mechanisms for S-ketamine compared with other treatments, only propofol, dexmedetomidine and sevoflurane groups were included. An overlapping effect of decreased metabolism was observed mainly in the thalamus bilaterally, and to lesser extent in the left anterior temporal fusiform gyrus (*p* < 0.05, FWE corrected at voxel level; Fig. 3*B*). The peak effect was located at the left central medial thalamic nucleus (CMT), while significant decreases were also observed in other adjacent thalamic nuclei.

Since the thresholds for state classification were defined *ad hoc*, we performed a confirmatory regression analysis using each subject's classification weight for unresponsiveness as an explanatory variable. These results largely converged with those observed using dichotomized states: in the propofol, dexmedetomidine and sevoflurane groups, disconnectedness was negatively correlated with CMR_glu_ mainly in the thalamus, while in the S-ketamine group there was a positive correlation between disconnectedness and CMR_glu_ in the left intraparietal sulcus and left angular gyrus (*p* < 0.05, FWE corrected at cluster level for propofol and sevoflurane groups and *p* < 0.001, uncorrected for dexmedetomidine and S-ketamine groups; Extended Data Fig. 3-1*A*). Conjunction analysis across propofol, dexmedetomidine and sevoflurane groups revealed overlapping negative correlation between disconnectedness and CMR_glu_ mainly in the thalamus bilaterally (*p* < 0.05, FWE corrected at voxel level; Extended Data Fig. 3-1*B*).

**Figure 4.**
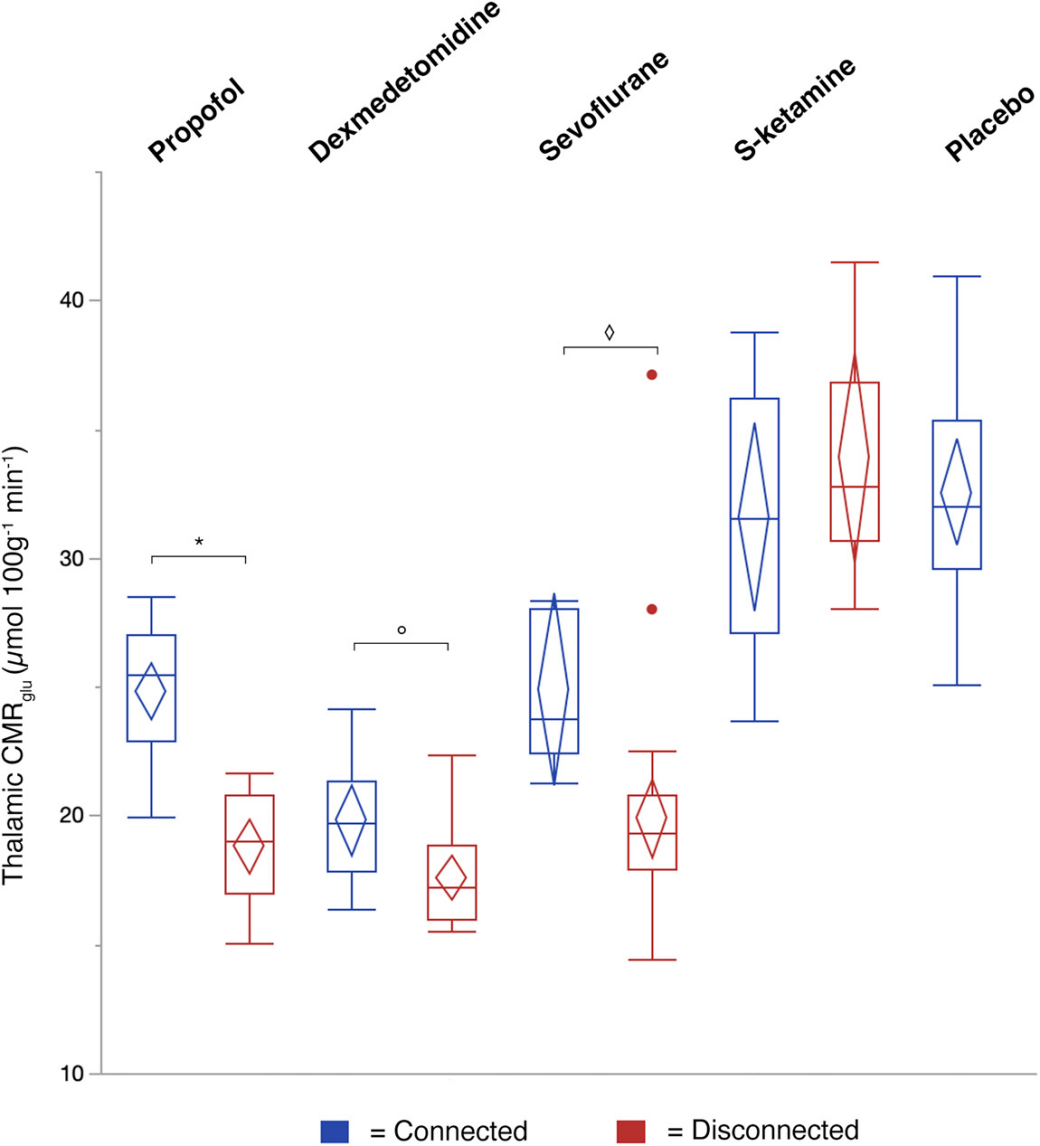
Glucose metabolism in the central medial thalamus in connected and disconnected states. Boxplots for absolute glucose metabolism at the peak effect location (central medial thalamic nucleus) from the conjunction analysis. Thalamic CMR_glu_ was significantly different between connectedness and disconnectedness in the propofol (*p < 0.0001), dexmedetomidine (°*p* = 0.0144) and sevoflurane groups (◊*p* = 0.0056). Boxes represent lower quartiles, medians and upper quartiles, and whiskers represent 1.5× interquartile ranges above and below the upper and lower quartiles, respectively. The middle of the confidence diamonds represents the mean, and the top and the bottom represent the upper and lower 95% confidence limits of the mean.

**Figure 5.**
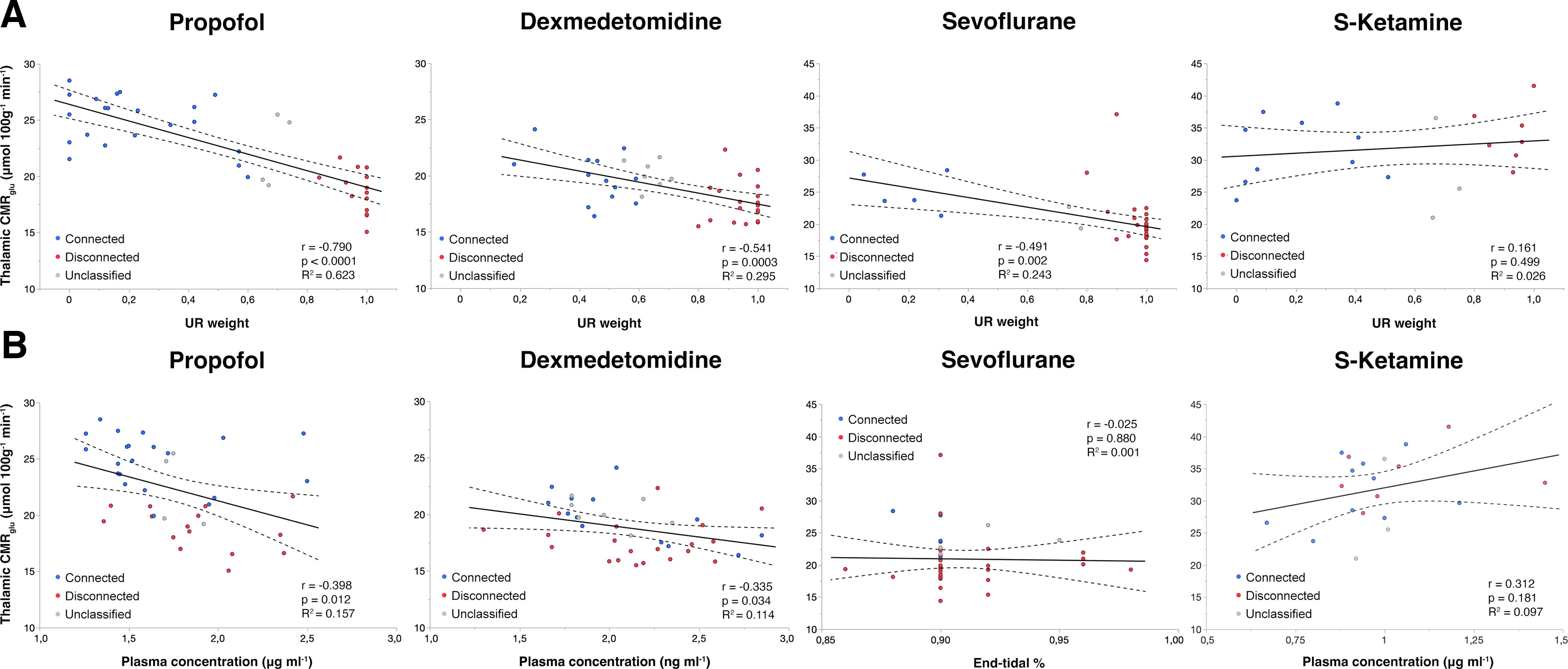
Thalamic glucose metabolism was more strongly correlated with the state of consciousness than with the measured drug concentration. ***A***, The estimated proportion of tracer uptake in the disconnected state (i.e., UR weight) was negatively correlated with glucose metabolism in the central thalamus in the propofol, dexmedetomidine and sevoflurane groups, but not in the S-ketamine group (*r*(38) = −0.790, *p* < 0.0001; *r*(38) = −0.541, *p* = 0.0003; *r*(38) = −0.491, *p* = 0.002 and *r*(18) = 0.161, *p* = 0.499, respectively). ***B***, Correlations between mean measured drug concentration and thalamic CMR_glu_ were weaker but statistically significant in the propofol and dexmedetomidine groups (*r*(38) = −0.398, *p* = 0.012 and *r*(38) = −0.335, *p* = 0.034, respectively). *r* = Pearson correlation coefficient, R^2^ = coefficient of determination, dashed lines depict the 95% confidence intervals of the regression lines.

Next, CMR_glu_ values were extracted within the thalamic peak effect location from the conjunction analysis (Fig. 3*B*) to explore absolute metabolic differences between connectedness and disconnectedness. In two-way ANOVA, there was a significant drug by state interaction (*F*_(3,113)_ = 7.2, *p* = 0.0002). In pairwise comparisons within drugs, the effect of state was significant in the propofol (*t*_(113)_ = 8.3, *p* < 0.0001), dexmedetomidine (*t*_(113)_ = 2.97, *p* = 0.0144 and sevoflurane (*t*_(113)_ = 3.26, *p* = 0.0056) groups, but not in the S-ketamine group (*t*_(113)_ = −1.01, *p* = 0.95) after Bonferroni correction (Fig. 4). There was a significant negative correlation between the estimated proportion of tracer uptake in the disconnected state and thalamic CMR_glu_ in the propofol, dexmedetomidine and sevoflurane groups (*p* ≤ 0.002), but not in the S-ketamine group (Fig. 5*A*). The drug concentrations were also negatively correlated with thalamic CMR_glu_ in the propofol and dexmedetomidine groups (*p* ≤ 0.034; Fig. 5*B*). However, the overall differences in drug concentrations in plasma explained relatively little of the variance in CMR_glu_ in comparison with the variance explained by the state of consciousness.

### Drug-related effects

**Figure 6.**
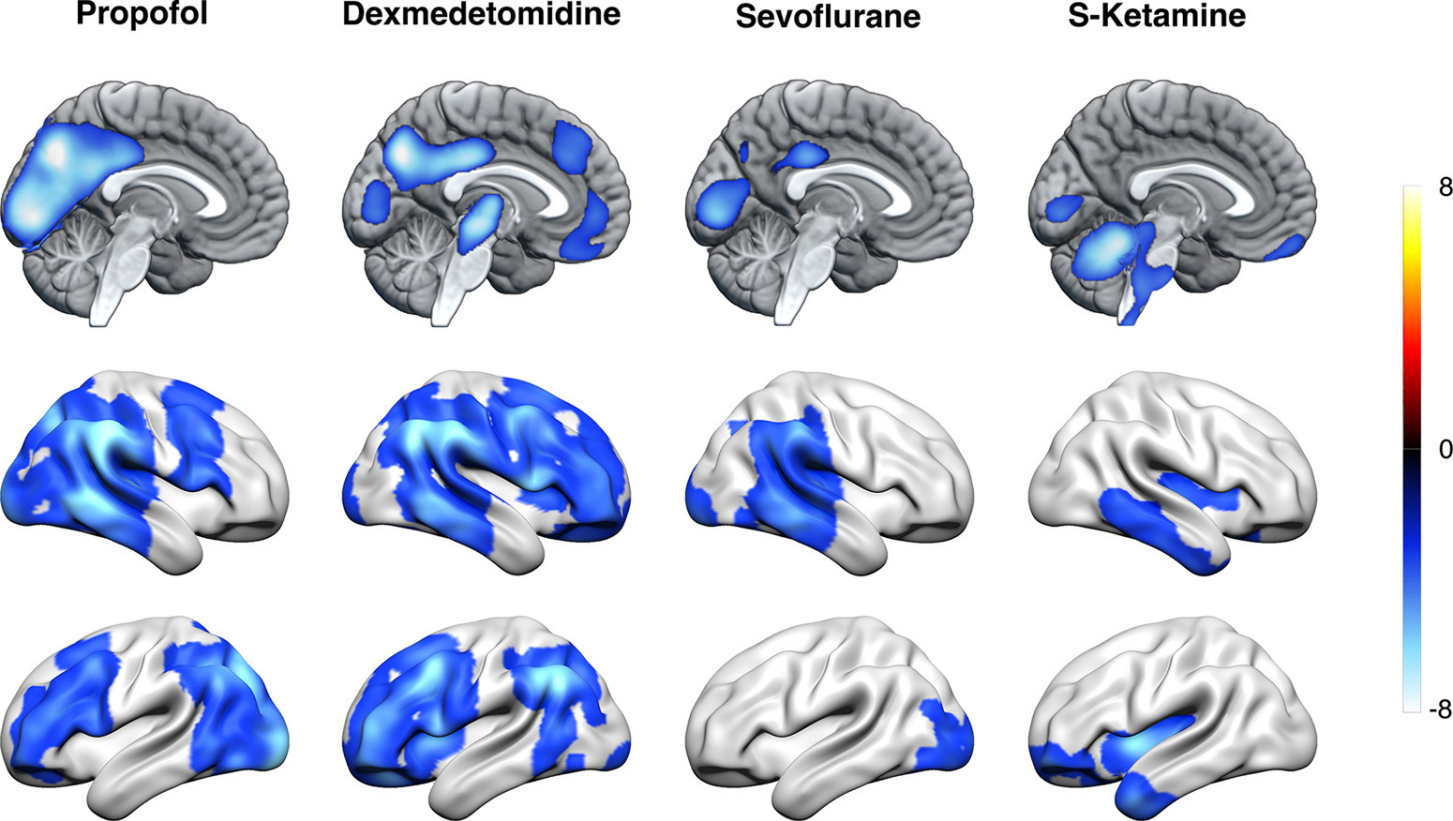
Drug-related effects. Relative rCMR_glu_ differences between placebo and connected subjects from each treatment group representing nonspecified drug-effects. Cool colors represent lower relative CMR_glu_ in the treatment groups. Propofol, dexmedetomidine and sevoflurane caused relative suppression of CMR_glu_ bilaterally in the posterior cingulate cortex, precuneus, inferior parietal cortex and occipital cortex. Additionally, propofol and dexmedetomidine induced suppression of CMR_glu_ bilaterally in the dorsolateral prefrontal cortex. Dexmedetomidine additionally suppressed CMR_glu_ bilaterally in the thalamus, dorsomedial prefrontal cortex, and ventromedial prefrontal cortex. S-ketamine induced relative decrease of glucose metabolism bilaterally in the insular cortex, ventromedial prefrontal cortex, occipital cortex, cerebellum, right inferior temporal cortex, left temporal pole, cerebellum, and brainstem areas. Statistical threshold set at *p* < 0.05 for all contrasts using FWE correction for multiple comparisons at cluster level, colorbar depicts *t* values.

To identify nonspecified drug-induced effects on brain activity, we contrasted each connected subjects' scans within each drug group with the placebo group scans. We found that exposure to propofol, dexmedetomidine or sevoflurane was associated with relative suppression of CMR_glu_ bilaterally in the posterior cingulate cortex, precuneus, inferior parietal cortex and occipital cortex (*p* < 0.05, FWE corrected at cluster level; Fig. 6). Propofol and dexmedetomidine induced additional suppression bilaterally in the dorsolateral prefrontal cortex. Dexmedetomidine additionally suppressed CMR_glu_ bilaterally in the thalamus, dorsomedial prefrontal cortex and ventromedial prefrontal cortex. Exposure to S-ketamine was associated with relative decreases of CMR_glu_ bilaterally in the insular cortex, ventromedial prefrontal cortex, occipital cortex, cerebellum, inferior temporal cortex, globus pallidus, nucleus accumbens, and left temporal fusiform cortex.

### Combined effects of drug and state

**Figure 7.**
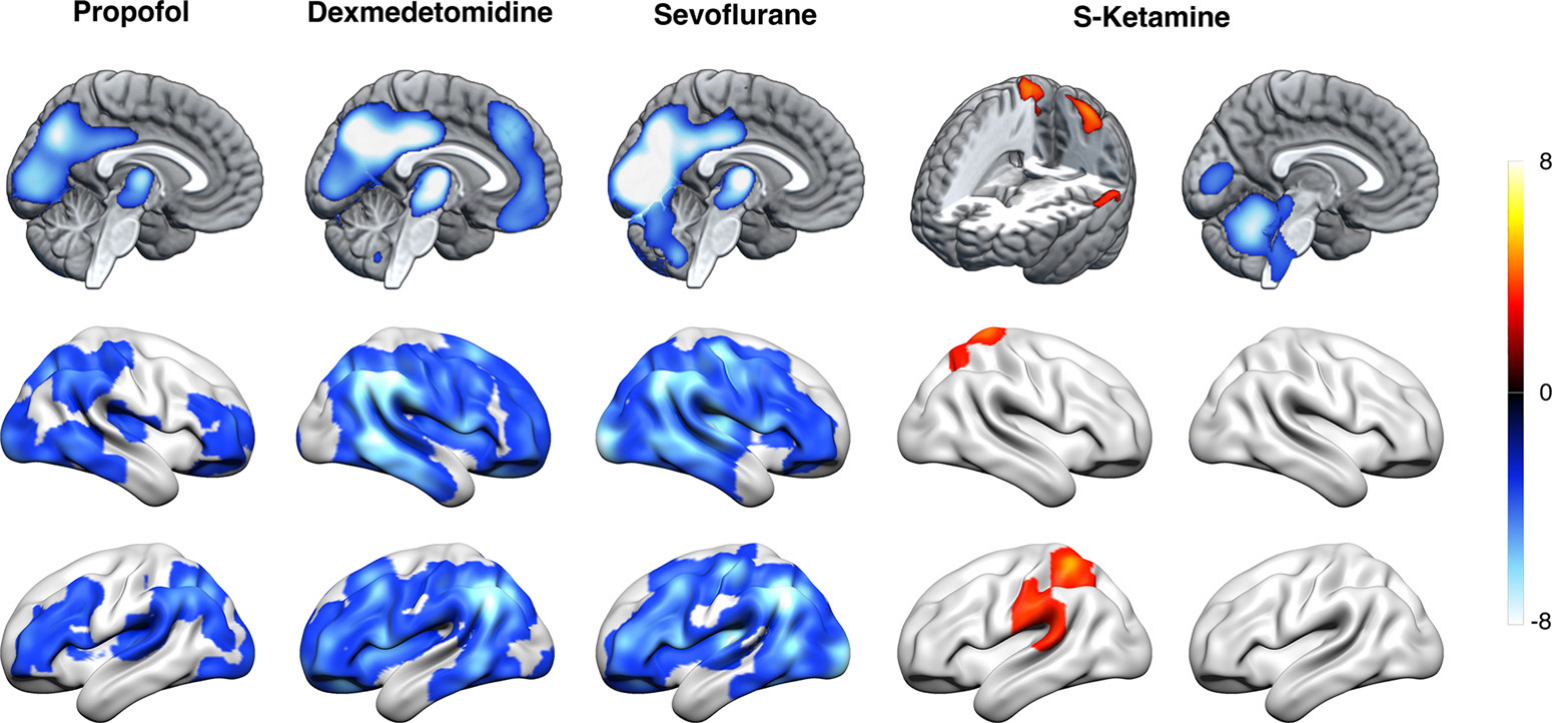
Combined effects of drug and state. Relative CMR_glu_ differences between placebo and disconnected subjects from each treatment group representing combined effects of drug and state. Cool colors represent lower, and warm colors higher relative CMR_glu_ in the treatment groups. Compared with the placebo group, disconnected subjects from propofol, dexmedetomidine and sevoflurane groups expressed a widespread pattern of reduced CMR_glu_ over bilateral cortical and subcortical areas. No significant increases in relative CMR_glu_ were observed for either propofol, dexmedetomidine or sevoflurane. In contrast, disconnected subjects in the S-ketamine group had relatively increased CMR_glu_ in left supramarginal gyrus and bilateral superior parietal lobule extending to right precuneal area (not visualized). Disconnected S-ketamine subjects also had lower CMR_glu_ in lingual gyrus, cerebellum and brainstem. Statistical threshold was at *p* < 0.05 for all contrasts using FWE correction for multiple comparisons at cluster level, colorbar depicts *t* values.

Contrasting each disconnected subjects' scans within each drug group with the placebo group scans, we found that propofol, dexmedetomidine and sevoflurane induced relative suppression of CMR_glu_ bilaterally in the thalamus, posterior cingulate cortex, precuneus, inferior parietal cortex, occipital cortex and dorsolateral prefrontal cortex (*p* < 0.05, FWE corrected at cluster level; Fig. 7). Dexmedetomidine additionally suppressed CMR_glu_ in the dorsal and ventral medial prefrontal cortices. Compared with placebo, S-ketamine induced relative increases in CMR_glu_ in the left supramarginal gyrus, bilateral superior parietal lobule and right precuneus. Relative CMR_glu_ decreases were noted bilaterally in the lingual and primary visual cortices, and in the brainstem, more specifically in the pons at the site of the locus coeruleus, and in the posterior aspect of the medulla.

## Discussion

We found that decreased metabolic activity of the thalamus was specifically associated with disconnectedness during EC_50_ administration of either propofol, dexmedetomidine or sevoflurane. Subjective experiences were frequently reported on awakening implicating that unresponsiveness denoted a state of disconnectedness but not necessarily unconsciousness. The specific effects related to the difference in the global state of consciousness were remarkably limited compared with the combined effects of the drug and the state, or the nonspecified drug-effects alone, which were both associated with much broader changes in brain activity in the frontal, parietal, temporal, and occipital cortical areas. Our findings also suggest that S-ketamine may differ from other investigated anesthetics not only in terms of the overall or nonspecified drug-effects, but also in terms of the specific mechanisms related to changes in the state of consciousness.

Several studies and theories of anesthetic mechanisms of action have indicated a critical role for the thalamus in regulating consciousness (Alkire et al., 2000; Alkire and Miller, 2005; John and Prichep, 2005; Ward, 2011; Mashour and Alkire, 2013). Early neuroimaging studies suggested that suppression of neuronal activity in the thalamus may represent a unified neural correlate for the change in the state of consciousness induced by several anesthetics with different primary mechanisms of action (Fiset et al., 1999; Alkire et al., 2000). In this view, the anesthetic action either directly or indirectly leads to hyperpolarization of thalamocortical neurons, switching their primary firing mode from tonic to burst firing (Alkire et al., 2000; Llinás and Steriade, 2006). Much like in nonrapid eye movement sleep, this would disrupt the information transfer in the thalamo-cortico-thalamic system leading to unawareness of external perceptual events. However, in the previous studies, the data comprised both the state-related and drug-related effects introducing a possible confound when interpreting the results. The current study resolves this shortcoming and specifically supports a role for the thalamus in regulating the state of consciousness.

Transitions between global states of consciousness during constant-dose anesthesia have previously been associated with brain activity changes in the thalamus, midline cortical areas and inferolateral parietal cortex (Xie et al., 2011; Långsjö et al., 2012; Scheinin et al., 2021). Because of FDG-tracer kinetics, the results of the current study reflect averaged brain activity over the 40-min steady-state drug exposure as opposed to the short period reflecting brain activity immediately after the state transition monitored with [^15^O]H_2_O perfusion imaging. We speculate that this is the principal reason why the results of the current study primarily pinpoint effects in the thalamus, as opposed to the broader network of brain regions revealed in the previous studies. This could implicate that while the activity of a network comprising the thalamus, anterior and posterior cingulate gyri and bilateral angular gyri associates with transitions between disconnected and connected states of consciousness within a shorter time frame (Scheinin et al., 2021), suppression of thalamic neuronal activity is crucial for preventing connected consciousness.

As cortical reactivity to sensory stimulation is relatively preserved in the primary sensory areas during anesthesia (Warnaby et al., 2016; Lichtner et al., 2018; Nourski et al., 2018), the current finding of suppressed thalamic activity is better understood as a mechanism contributing to disrupted thalamo-cortico-thalamic information processing (Alkire et al., 2008) rather than a switch preventing information from reaching the cortex in the first place. This view is in agreement with both empirical findings and computational models of altered thalamocortical interactions during anesthesia and sleep (White and Alkire, 2003; Hill and Tononi, 2005; Boveroux et al., 2010; Liu et al., 2013; Vijayan et al., 2013). Supporting this view, we found the peak suppression of thalamic metabolism at the CMT and adjacent medial thalamic nuclei, which have diffuse projections to the cortex and striatum, and are associated with modulation of cortical activity patterns, arousal and cognitive functions (Saalmann, 2014; Schmitt et al., 2017). Consistently, reversal of general anesthesia has been accomplished by CMT microinfusion of either nicotine (Alkire et al., 2007) or a specific antibody blocking a type of voltage-gated potassium channels (Alkire et al., 2009). Recently, electrical stimulation of rostral intralaminar thalamic nuclei has been shown to promote arousal in anesthetized macaques while shifting cortical low-frequency and high-frequency power, spiking and coherence (Redinbaugh et al., 2020; Bastos et al., 2021), and static and dynamic functional connectivity (Tasserie et al., 2022) toward awake baseline dynamics. Furthermore, bilateral lesion of intralaminar thalamic nuclei has been associated with impairment of consciousness (Långsjö et al., 2016).

Our findings with propofol and sevoflurane suggest that preferential cortical suppression, as witnessed in sedated but responsive subjects, precedes the transition from a connected to a disconnected state. This likely reflects a combination of direct and indirect (mediated through inhibition of the arousal nuclei) cortical drug-effects. Instead, preferential suppression of the thalamus was specifically associated with disconnectedness. With dexmedetomidine, we found the thalamus to be preferentially suppressed already in responsive subjects, possibly reflecting inhibition of arousal pathways through α_2_-adrenoceptor activation-mediated disinhibition of the ventrolateral preoptic area. This view is supported by Baker et al. (2014), who reported increased δ power in CMT and cortex with dexmedetomidine before loss of righting reflex in rats. Consistently, subjects classified as connected were less responsive during the tracer uptake in the dexmedetomidine group compared with other treatments based on median uptake weights. This may explain why the difference in thalamic activity between connectedness and disconnectedness was smaller with dexmedetomidine than with propofol or sevoflurane.

Exposure to S-ketamine was not associated with similar state-related metabolic effects as with other agents. The dissociative state characteristic of (S-)ketamine is likely caused by preferential antagonism of NMDA receptors in GABAergic interneurons leading to disinhibition of excitatory pyramidal neurons (Homayoun and Moghaddam, 2007; Vollenweider and Kometer, 2010). Consistently, we found a weak state-related effect of increased metabolic activity in left intraparietal sulcus, which is in agreement with previously reported metabolic effects of anesthetic concentrations of S-ketamine (Långsjö et al., 2005). The high metabolism and complexity with ketamine and low metabolism and complexity with other anesthetics has often been associated with their respective high and low probabilities for reporting disconnected conscious experiences on awakening (Lee et al., 2022). This has likely contributed to the common assumption that high metabolism and complexity are necessary preconditions for consciousness. In the current study, disconnected consciousness was frequently reported after low metabolism brain states, challenging the notion that the difference in the observed brain states between ketamine and other anesthetics could be simply related to high or low awareness. While the interpretations related to the neural correlates of consciousness *per se* are out of the scope of the current study, our findings support the view that disconnectedness can be caused by low and high metabolism mechanisms.

A notable limitation of the current study is that the interviews used for verifying disconnectedness were only obtained at the end of the experiment. Report analysis suggested profound amnesia since awareness of external events was only reported by one responsive subject from the S-ketamine group (Radek et al., 2021) despite frequent signs of connected consciousness implicated by responses to auditory ques. However, based on our previous experiments using serial awakenings with immediate interviews, explicit awareness is rare in unresponsive subjects although internal subjective experiences may still occur rather frequently (Noreika et al., 2011; Radek et al., 2018; Valli et al., 2023). The use of a face mask for sevoflurane administration may have caused relative stimulation in comparison to intravenous anesthetics. While this could have influenced between-group comparisons, it is unlikely that the main within-group results were affected. Furthermore, the proportion of disconnected subjects was higher in the sevoflurane group compared with other treatments. Generally, there were more unresponsive than responsive trials, suggesting that we slightly overestimated the EC_50_ doses. This was especially evident in the sevoflurane and dexmedetomidine groups.

The frequently repeated testing for responsiveness revealed important information about the labile nature of the state of consciousness at the estimated EC_50_ anesthetic exposure. While the state fluctuation within subjects could have reduced the sensitivity to detect state-related differences between subjects, it allowed us to test for associations between the degree of disconnectedness and brain activity. The convergence of the results between the two analysis methods suggests that the effect of the within-subject state fluctuation on the main finding was relatively small.

The most notable strength of the study is the use of four anesthetics with different primary mechanisms of action in the same experimental settings while allowing distinction between the state-related and drug-related effects. In summary, we found consistent evidence that decreased thalamic activity is a specific correlate of disconnectedness for three commonly used anesthetics representing three different primary mechanisms of action. Our results highlight the need for carefully designed studies to elucidate the neural mechanisms associated with specific states of consciousness.
