# Supplementary figures and images for: Decreased Thalamic Activity Is a Correlate for Disconnectedness during Anesthesia with Propofol, Dexmedetomidine and Sevoflurane But Not S-Ketamine
Source: J Neurosci. 2023 Jun 28;43(26):4884–95. doi: 10.1523/JNEUROSCI.2339-22.2023 (PMC10312059; doi:10.1523/JNEUROSCI.2339-22.2023)

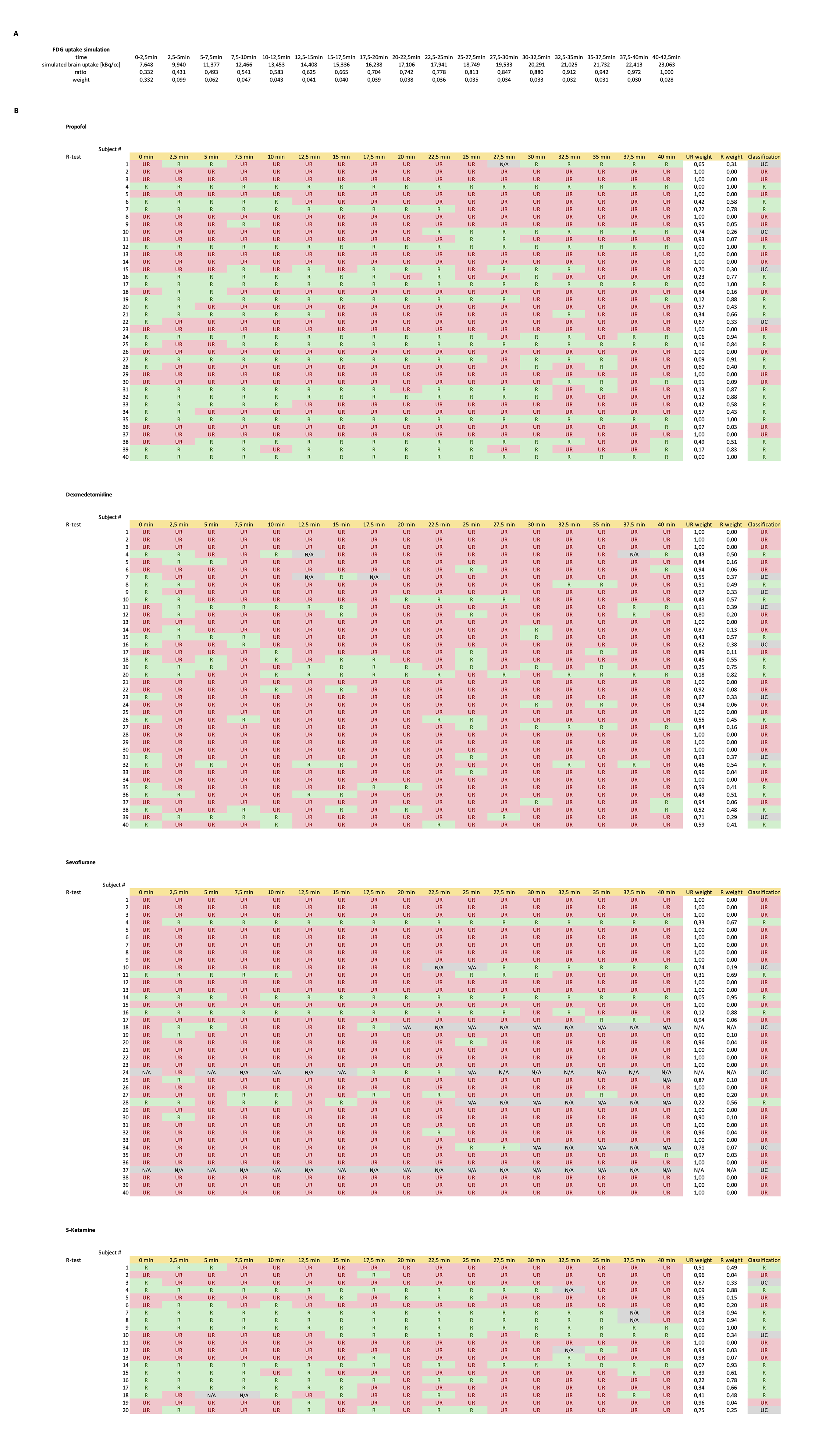

Supplement: Extended Data Figure 2-1 — Responsiveness weights based on tracer kinetic modeling and summary of responsiveness during the experiment. A, Tracer kinetic modeling was used to estimate cumulative [18F]FDG brain uptake for each 2.5-min R-test period. Assuming stabilized brain retention of the [18FFDG tracer by the time of the last R-test, cumulative tracer uptake ratios were calculated for each R-test period by dividing the simulated uptake value at respective time frames with the total tracer uptake value. Weights were then obtained by calculating the increase in cumulative uptake ratio for every sequential R-test period, reflecting their proportional significance in forming the final CMRglu image. B, R-test results in each study subject measured at 2.5-min intervals. The weights were combined with R-test results to estimate total [18FFDG brain uptake in either a responsive or an unresponsive state. Final weights and the ensuing subject classification (UR, R, or UC) are indicated in the three columns to the right. UR = unresponsive, R = responsive, UC = unclassified. Download Figure 2-1, TIF file. [file ns-JN-RM-2339-22-s02.tif]

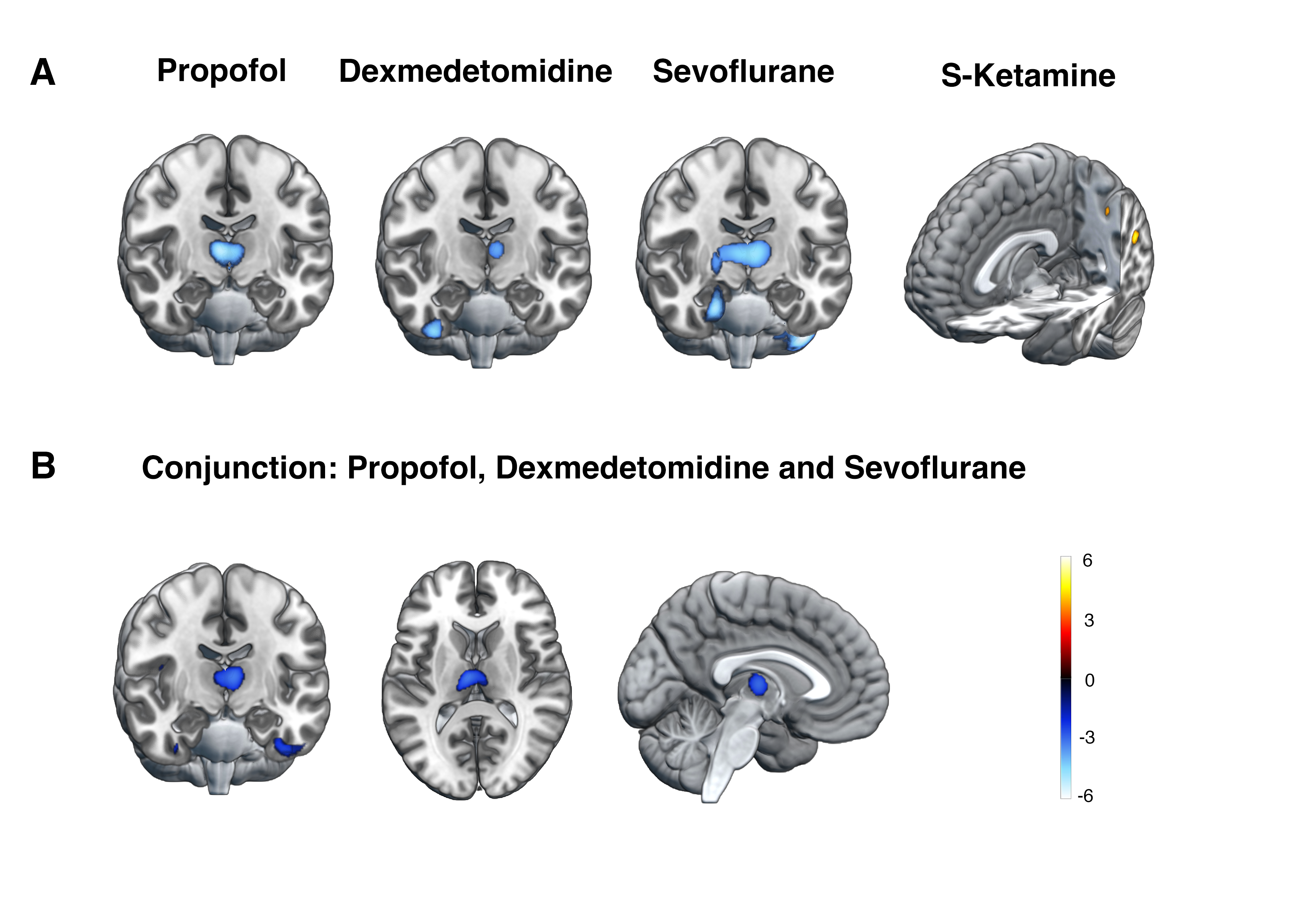

Supplement: Extended Data Figure 3-1 — Relationship between disconnectedness and CMRglu. A, In the propofol group, disconnectedness was negatively correlated with CMRglu bilaterally in the thalamus (p < 0.05, FWE corrected at cluster level) In the sevoflurane group, similar correlations were found bilaterally in the thalamus, right medial temporal cortex, and the cerebellum. In the dexmedetomidine and S-ketamine groups, state-metabolism correlations were found only at a significance threshold of p < 0.001, uncorrected. In the dexmedetomidine group, disconnectedness was negatively correlated with CMRglu in the left medial thalamus and ventral posterior cingulate cortex, while in the S-ketamine group, there was a positive correlation between disconnectedness and CMRglu at the intersection of the left intraparietal sulcus and dorsal angular gyrus and in the left rostroventral angular gyrus. B, Conjunction analysis across propofol, dexmedetomidine and sevoflurane groups revealed overlapping negative correlation between disconnectedness and CMRglu mainly bilaterally in the thalamus, and to lesser extent bilaterally in the inferior temporal cortices and dorsal insular cortex (p < 0.05, FWE corrected at voxel level). The colorbar depicts t values. Download Figure 3-1, TIF file. [file ns-JN-RM-2339-22-s01.tif]
